# Amniotic Membrane Granuloma in a Case of Ocular Chemical Injury: Clinical Features, Histopathology, and Outcomes

**DOI:** 10.7759/cureus.19171

**Published:** 2021-10-31

**Authors:** Anahita Kate, Sayan Basu

**Affiliations:** 1 Cornea, L V Prasad Eye Institute, Vijayawada, IND; 2 Cornea, L V Prasad Eye Institute, Hyderabad, IND

**Keywords:** ocular surface, simple limbal epithelial transplant, chemical injury, granuloma, amniotic membrane

## Abstract

This report describes the clinical features of a granuloma within the layers of amniotic membranes (AM) along with its surgical management and outcome. An eight-year-old boy underwent an AM graft in the right eye for an acute chemical injury. As there was slow healing of the ocular surface, the eye was subjected to a simple limbal epithelial transplantation (SLET). Although the corneal surface was well epithelialized, a superior symblepharon was noted six weeks following SLET. Excision of the fibrotic tissue with an AM graft was carried out. Growth of a grayish-white fibrotic lesion in the visual axis of the cornea was noted eight months after the second AM graft. The optical coherence tomography line scan revealed the location of the lesion to be anterior to the retained SLET AM. This lesion progressively increased in size, so an excision biopsy was carried out. The lesion excised in toto with the AM, and an optically clear plane was noted. Histopathology of the tissue revealed the presence of myofibroblasts, which possibly originated from the AM fibroblasts. At the three-year follow-up period, there was no recurrence of the growth, and the final visual acuity was 20/40. This is a rare report of a granuloma case arising within the layers of an AM. The underlying etiopathogenesis could be due to the multiple AM grafts that the patient underwent. These repeated grafts can incite an immune response and lead to the formation of a granuloma. The special staining and the restoration of the corneal clarity with a stable ocular surface suggest the AM origin of the mass lesion. Removal of the layer of the AM with the granuloma has good outcomes, with no recurrence on long-term follow-up.

## Introduction

The role of an amniotic membrane (AM) in a case of chemical injury is multimodal. In the acute phase, the AM helps the healing process through its anti-inflammatory and anti-fibrotic properties and by promoting surface re-epithelialization [1,2]. In the chronic phase, it assists in surface reconstruction by acting as a scaffold for both conjunctival and corneal epithelial cells [3]. Usually, cases of ocular burns require repeated AM grafts during the course of healing. Although the AM inherently suppresses various immune responses, the grafting of multiple layers over a period of time can trigger an immune response against the AM within the host tissue [4]. This can lead to aggregates of inflammatory cells within the AM. This report details the clinical features and management of one such case, wherein the inflammatory cells within the AM lead to the formation of a granuloma. The outcome of iatrogenic de-epithelialization of the cornea following a limbal stem cell transplant is also described within the report.

## Case presentation

**Figure 1:**
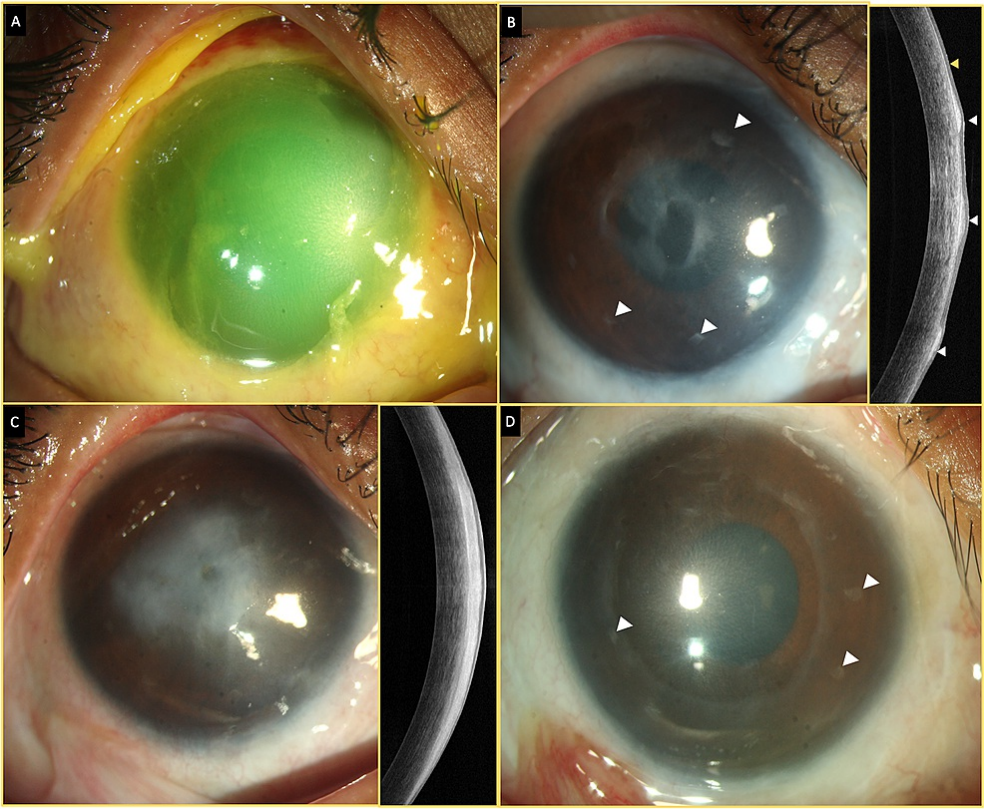
A collage of images that depicts the clinical course of the patient (A) Slit-lamp photograph of the right eye showing a total corneal defect taking up fluorescein stain. The limbus appears ischemic in all quadrants. (B) Three months following simple epithelial limbal transplantation (SLET), a stable ocular surface is present with intact SLET implants (white arrowheads). A 4.5 x 3 mm grayish-white lesion with ill-defined margins is seen in the visual axis. The optical coherence tomography line scan shows the lesion to be hyperreflective, irregular (white arrowheads), and superior to the plane of the amniotic membrane (yellow arrowhead). (C) An increase in the density and size of the lesion is seen eight months after the presentation. (D) Clinical image after excision biopsy showing no recurrence of the lesion with intact implants (white arrowheads) and a stable epithelial surface.

An eight-year-old boy with acute chemical injury presented to the emergency clinic at our institute with a history of a fall of limestone in the right eye three days prior to presentation. There was redness, pain, and decreased vision since then. The visual acuity at the presentation was 20/320. On examination, there was significant lid edema, a superior conjunctival epithelial defect, and a total corneal epithelial defect (Figure 1, Panel A). The limbus appeared ischemic in all quadrants (Figure 1, Panel A). The left eye was uninvolved and had an unaided vision of 20/20. The child was examined under anesthesia, and following a thorough wash of the right eye with balanced salt solution, the retained foreign bodies within the upper and lower lids were removed. An AM was secured over the palpebral and bulbar conjunctiva with fibrin glue (Tisseel Kit, Baxter AG, Vienna, Austria), and a permanent lateral paramedian tarsorrhaphy was done with 6-0 Vicryl suture (Ethicon Inc., Ohio, USA).

Postoperatively, the patient was started on hourly prednisolone acetate 1%, prophylactic antibiotics (moxifloxacin 0.5% four times/day), and carboxymethylcellulose 0.5% (eight times/day). Ten days after the initial presentation, a large persistent epithelial defect was noted under the AM. In view of the extensive involvement and the non-healing ocular surface, the patient was planned for a simple limbal epithelial transplantation (SLET). The surgical procedure was carried out as per the standard protocol [5]. In brief, the limbal biopsy was harvested from the superior limbus of the left eye along with a separate conjunctival autograft measuring five clock hours. After doing a 360-degree conjunctival peritomy and dissecting the tenons, 2-3 mm of the bare sclera was exposed, and an AM with epithelial side up was draped over the corneal surface with fibrin glue and tucked underneath the free margins of the conjunctiva. The biopsy tissue pieces were secured over the layer of AM with fibrin glue, and the conjunctival autograft was placed superiorly after excising an area of necrotic conjunctiva.

Postoperatively, the patient was started on a tapering dose of topical steroids (prednisolone acetate 1%, started at a dose of six times/day, which was gradually tapered every five days) and prophylactic antibiotics (moxifloxacin 0.5% four times/day). The patient was followed up closely, and complete re-epithelialization of the corneal surface was noted within three weeks of the procedure. Six weeks following SLET, there was a formation of a superior symblepharon with fibrotic constriction, and to address this, excision of the fibrotic tissue with an AM graft was carried out. The AM was draped over the cornea and the superior bare scleral area and secured with fibrin glue. Following the surgery, the patient was given a tapering course of steroids with antibiotics similar to the regimen mentioned above.

**Figure 2:**
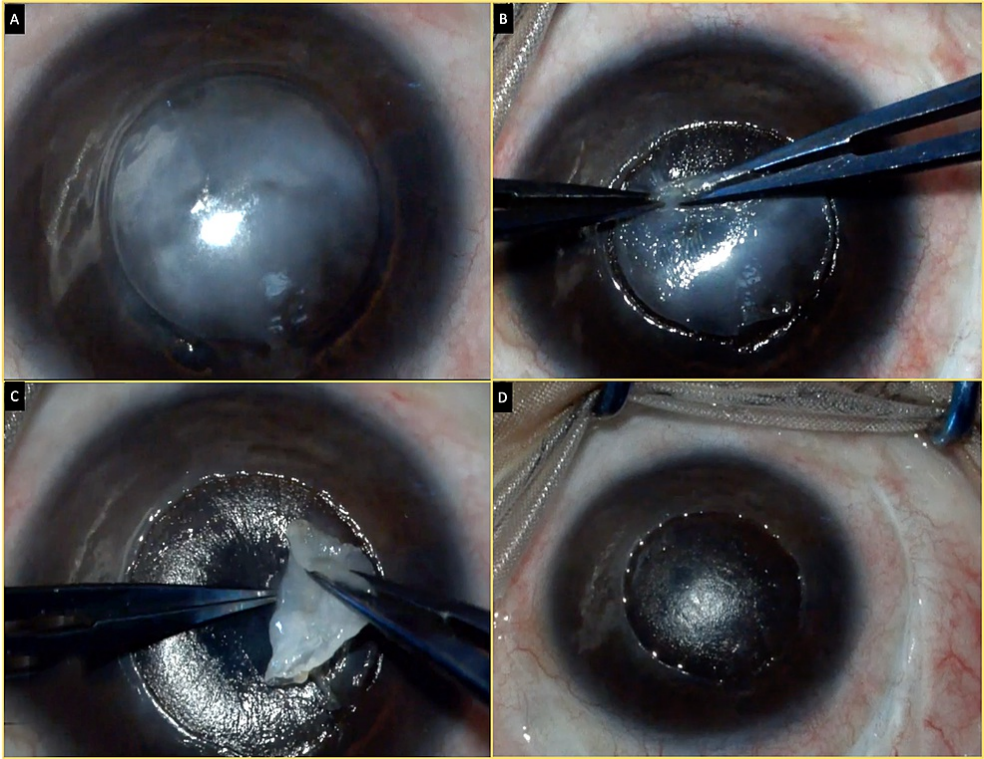
A collage of images illustrating the intraoperative steps (A) The margins of the lesion are delineated with a 7-mm corneal trephine. (B and C) The plane of the lesion with the retained amniotic membrane is identified, and the complex is manually dissected and excised. (D) An optically clear plane has been obtained with no macroscopic residual lesions.

Six weeks after this procedure, the child presented with an unaided vision of 20/100. On slit-lamp examination, a 4.5 x 3 mm grayish-white lesion with ill-defined margins was noted in the subepithelial plane of the cornea in the visual axis. The surrounding cornea was uninvolved, and the SLET implants were intact. The rest of the eye was quiet (Figure 1, Panel B). The optical coherence tomography (OCT) line scan revealed the lesion to be hyperreflective, irregular, and anterior to the retained SLET AM (Figure 1, Panel B). A progressive increase in the size and density of the lesion was noted over the subsequent months, and after eight months of follow-up, the lesion obscured the visual axis with a drop in vision to 20/800 (Figure 1, Panel C). Excision of the mass lesion was planned, and intraoperatively, after delineating the margins of the lesion with a 7-mm corneal trephine, manual blunt dissection was carried out to identify the plane of the AM (Figure 2, Panels A-C). Following this, the layer was peeled off leaving behind bare stroma that appeared optically clear (Figure 2, Panel D). A bandage contact lens was placed over the cornea. Postoperatively, the patient was started on antibiotics and a tapering course of steroids.

**Figure 3:**
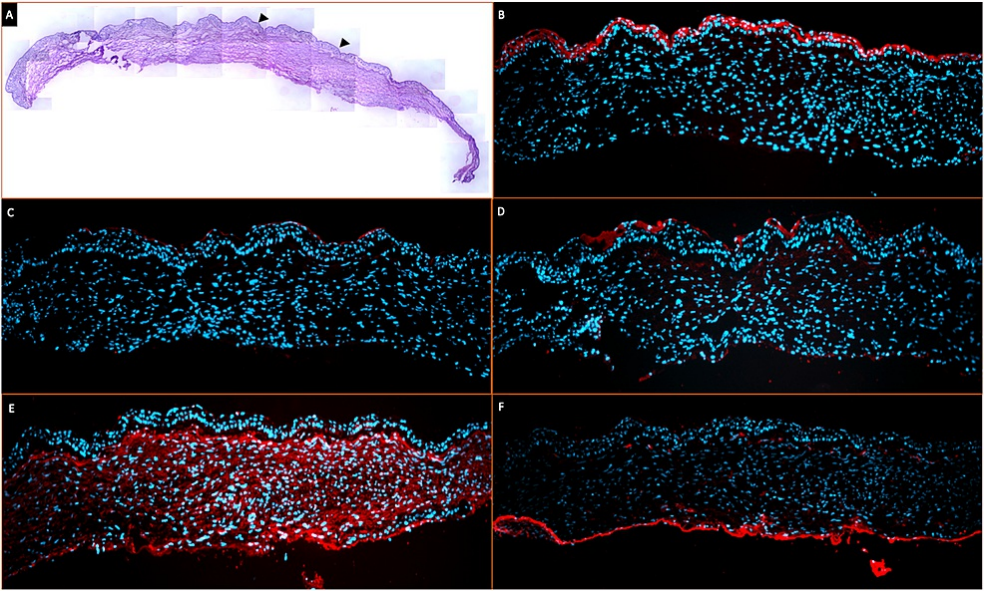
A collage of histopathology and immunohistochemistry images (10x magnification) (A) A montage image of the entire excised mass lesion. A continuous stratified epithelial layer (black arrowheads) is noted with a loose matrix dispersed with chronic inflammatory cells. (B) On immunohistochemistry staining, the epithelium was negative for CK19 and positive for CK3. (C and D) The presence of myofibroblasts is demonstrated by the smooth muscle actin (SMA) positivity. (E) The lesion is strongly positive for vimentin. (F) Positive staining for collagen type III in the basal region of the lesion.

The excised tissue was subjected to histopathological examination, wherein a continuous stratified epithelial layer was noted with a loose matrix dispersed with chronic inflammatory cells (Figure 3, Panel A). On immunohistochemistry staining, the epithelium was positive for CK3 and negative for CK19 (Figure 3, Panels B and C). The lesion was also strongly positive for vimentin, and the presence of myofibroblasts was demonstrated by the smooth muscle actin (SMA) positivity (Figure 3, Panels D and E). Lastly, there was positive staining for collagen type III in the basal region of the lesion (Figure 3, Panel F).

There was no recurrence of the fibrotic lesion on subsequent follow-up visits, and a stable ocular surface was noted (Figure 1, Panel D). The patient developed posterior subcapsular cataract six months after the excision of the lesion, for which he underwent phacoemulsification with an intraocular lens. Raised intraocular pressure was noted for which the patient was started on topical antiglaucoma medications (dorzolamide 2% + timolol 0.5%). Glaucomatous disc damage with a cup disc ratio of 0.7 was present. The corrected vision at the last follow-up which was three years after the initial presentation was 20/40 and 20/30 for distance and near, respectively. The eye had a stable ocular surface with intact SLET implants. The intraocular pressure was well controlled with the antiglaucoma medications with no progression of disc damage.

## Discussion

Traditionally an AM is considered to be immunosuppressive in lieu of its anti-inflammatory and anti-angiogenic properties [3]. However, under a favorable milieu, the AM can switch from an immunosuppressive implant to an immunostimulant one causing T cell proliferation and macrophage infiltration [6]. Since it lacks the necessary human leukocyte antigen (HLA) proteins, its allogenic origin is not recognized by the body, and so no immune response is mounted against it [3]. Nevertheless, while the latter may hold true for first-time AM transplants, there are reports of repeated grafts inducing an immune reaction within the host tissue against the donor. Gabler and Lohmann have hypothesized that receiving multiple grafts from the same donor increases the risk of eliciting an immune reaction [4]. In our case, there were multiple transplants of AM, which could have triggered the formation of the granuloma. These grafts were from different donors, suggesting that the activation of an immune response may depend on a load of foreign antigens or the stimulus may be a non-specific factor present commonly in all AMs setting off an inflammatory cascade in susceptible individuals.

The immunohistochemistry (IHC) staining of the excised tissue provides an insight into its origin. The CK3 positivity and lack of CK19 staining indicate the corneal origin of the epithelium and the functioning of the SLET implants [7]. During the course of normal wound healing, there occurs migration of fibroblasts, which in the presence of inflammatory mediators and cytokines differentiate into proto-myofibroblasts that then mature into myofibroblasts [8,9]. The presence of myofibroblasts in the current case is demonstrated by both the SMA and vimentin staining [8,10]. The former is present only in mature myofibroblasts, while the latter is an intermediate filament protein within the myofibroblast. Although the myofibroblasts are a part of the normal wound healing response and undergo apoptosis once the response is complete, an imbalance within this process leads to persistence of these cells, which then produces an abundance of extracellular matrix and contractile elements that lead to scar contracture [8,10]. The source of these myofibroblasts in our patient could have been from the keratocyte-derived fibroblasts or from the fibroblasts within the AM. The presence of type III collagen in the basal region of the tissue indicates the presence of either the retained AM or the Bowman's layer of the cornea as both structures contain type III collagen [3,11]. This delineating layer decreases the likelihood of a keratocyte-derived fibroblast migrating above the AM. Also, the presence of an intact basement membrane (BM) of the corneal epithelium suggests that the myofibroblasts developed from the AM fibroblast as an intact BM inhibit the conversion of keratocyte-derived fibroblasts into myofibroblasts [9]. These findings together indicate that the fibrotic lesion was likely to have originated from the retained SLET AM.

In the current case, the excision of the granuloma with the AM resulted in a large area of iatrogenically de-epithelialized cornea. This area healed over a course of two weeks, and the surface continued to be stable until the end of the follow-up period. This highlights the potential of the transplanted limbal stem cells to not only maintain the constant turnover of epithelial cells needed to sustain a normal corneal surface but also to re-epithelialize a damaged or denuded area. Also, these smaller islands of stem cells are able to cope with the increased demand of re-establishing a stable surface over a de-epithelialized area in a time frame similar to what was accomplished by the stem cells within a completely healthy limbus [12,13].

## Conclusions

This case describes the clinical features and outcomes of an unusual case of a granuloma arising from an amniotic membrane. The patient had undergone repeated grafts of the AM, which is a potential risk factor for inciting an inflammatory response against the AM. The histopathology in the current case with mature myofibroblasts indicates the chronicity of the lesion. Although the exact origin of these cells is unclear, the special staining with positivity for vimentin and an intact collagen type III layer beneath the lesion suggests that the lesion could have originated from the AM. In our patient, there was no recurrence of the growth, and a stable, optically clear corneal surface was maintained with the limbal stem cell transplantation until the three-year follow-up period. This report thus highlights the need for awareness of such an entity in cases wherein multiple AM grafts have been carried out. By confirming the subepithelial location of the lesion with an OCT, a simple excision of the lesion can be performed, which is associated with good outcomes.
